# Non-Coding RNAs in Cancer Radiosensitivity: MicroRNAs and lncRNAs as Regulators of Radiation-Induced Signaling Pathways

**DOI:** 10.3390/cancers12061662

**Published:** 2020-06-23

**Authors:** Marta Podralska, Sylwia Ciesielska, Joost Kluiver, Anke van den Berg, Agnieszka Dzikiewicz-Krawczyk, Izabella Slezak-Prochazka

**Affiliations:** 1 Institute of Human Genetics, Polish Academy of Sciences, 60-479 Poznań, Poland; marta.podralska@igcz.poznan.pl; 2 Department of Systems Biology and Engineering, Faculty of Automatic Control, Electronics and Computer Science, Silesian University of Technology, 44-100 Gliwice, Poland; sylwia.ciesielska@polsl.pl; 3 Department of Pathology and Medical Biology, University of Groningen, University Medical Center, Groningen, 9700RB Groningen, The Netherlands; j.l.kluiver@umcg.nl (J.K.); a.van.den.berg01@umcg.nl (A.v.d.B.); 4 Biotechnology Centre, Silesian University of Technology, 44-100 Gliwice, Poland

**Keywords:** non-coding RNA, miRNA, lncRNA, circRNA, radiation response, radiotherapy

## Abstract

Radiotherapy is a cancer treatment that applies high doses of ionizing radiation to induce cell death, mainly by triggering DNA double-strand breaks. The outcome of radiotherapy greatly depends on radiosensitivity of cancer cells, which is determined by multiple proteins and cellular processes. In this review, we summarize current knowledge on the role of microRNAs (miRNAs) and long non-coding RNAs (lncRNAs), in determining the response to radiation. Non-coding RNAs modulate ionizing radiation response by targeting key signaling pathways, including DNA damage repair, apoptosis, glycolysis, cell cycle arrest, and autophagy. Additionally, we indicate miRNAs and lncRNAs that upon overexpression or inhibition alter cellular radiosensitivity. Current data indicate the potential of using specific non-coding RNAs as modulators of cellular radiosensitivity to improve outcome of radiotherapy.

## 1. Introduction

Radiation therapy is a common treatment for many types of cancer, either alone or in combination with other therapeutic approaches. Different tumor types vary in their sensitivity to radiotherapy [1]. The response to radiation treatment is mediated by cellular processes such as DNA damage repair, apoptosis, cell cycle redistribution, etc., and some tumors exhibit high level of radioresistance. For an effective treatment, patients with radioresistant tumors require higher doses of irradiation which will lead to undesired side effects. Identification of factors leading to radioresistance and markers of radiosensitivity is crucial for identifying responsive patients and improving the outcome of radiotherapy. Recently, it has been demonstrated that expression patterns of non-coding RNAs (ncRNAs) after ionizing radiation (IR) differ between radioresistant and radiosensitive tumors [2,3,4,5,6,7].

Non-coding RNAs are a diverse group of functional RNA molecules that are not translated into proteins. Non-coding RNAs are predominant in the genome and can be divided into housekeeping ncRNAs, such as transfer RNAs (tRNA) and ribosomal RNAs (rRNA), and regulatory ncRNAs, which can be divided into small (<200 nt) ncRNAs, including microRNAs (miRNAs), and long (>200 nt) ncRNAs (lncRNAs) [8]. miRNAs are single-stranded, ~22 nt long ncRNAs that bind to a short core sequence mainly in the 3′ untranslated region (UTR) of target messenger RNA (mRNA) and lead to inhibition of translation or mRNA degradation [9,10]. lncRNAs are the largest class of ncRNAs with currently a total of >90,000 annotated genes [11]. A subset of the lncRNAs are circular RNAs (circRNAs) that are formed during splicing of linear transcripts (both protein coding and non-coding) into covalently closed single-stranded RNA molecules [12]. Regulation of gene expression by lncRNAs is more complex as they can interact with DNA, RNA, or proteins and act as transcriptional modulators, RNA splicing regulators, microRNA sponges, RNA-binding protein (RBP) sponges, and scaffolds to form ribonucleoprotein complexes [13,14,15]. Aberrant miRNA and lncRNA expression signatures have been reported for most cancer types and some of these ncRNAs were shown to impact on cellular functions such as cell proliferation and resistance to apoptosis.

Current studies revealed several mechanisms through which ncRNAs contribute to response to radiotherapy. Here, we summarize the main findings on ncRNAs involved in crucial pathways in response to radiotherapy in cancer. We focused on miRNAs and lncRNAs that regulate DNA double strand breaks (DSBs) repair, apoptosis, autophagy, cell cycle progression, glycolysis and Wnt/β-catenin pathway in response to IR. We also present an overview of ncRNAs that alter radiosensitivity of cancer cells (Table 1 and Table 2) and discuss the utilization of ncRNAs to improve the outcome of radiotherapy.

## 2. ncRNAs Involved in DSB Repair

**Figure 1:**
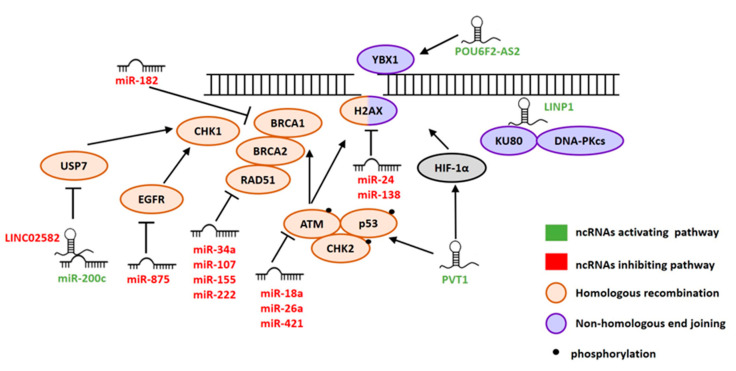
Non-coding RNAs (ncRNAs) regulating ionizing radiation (IR)-induced DSB repair. ncRNAs promoting (green) or inhibiting (red) DSB repair. The color of proteins indicates main pathways of DSB repair.

DNA double strand breaks (DSBs) are the most prevalent and deleterious type of damage induced by IR. If not repaired correctly, it can lead to cell death or chromosomal aberrations and genomic instability. Cells use two major mechanisms to repair DSBs, the non-homologous end joining (NHEJ) and homologous recombination (HR) pathways. The NHEJ pathway ligates broken DNA ends directly, whereas HR employs a homologous DNA template from the sister chromatid [138]. Both pathways require several proteins, which cooperate to detect (ATM, ATR, CHK1, CHK2), signal (γH2AX) and repair DSBs (BRCA1, BRCA2, Ku80, DNA-PKcs) [139,140]. Recently, the role of ncRNAs in DSB repair has been recognized [141]. Here, we summarize ncRNAs involved in DSB repair following IR (Figure 1).

### 2.1. miRNAs

Two miRNAs, miR-24, and miR-138 were shown to directly regulate H2AX upon IR. Phosphorylated H2AX (γH2AX) foci are early markers of DSBs, since H2AX recruits DNA repair proteins to DSB damage sites [142]. Overexpression of miR-24 decreased H2AX levels resulting in a diminished capacity to repair DSBs upon γ-irradiation in differentiated leukemic cells. DSB repair was restored by overexpressing a miR-24-insensitive *H2AX* gene [23]. A similar effect was shown in osteosarcoma cells where miR-138 mimics reduced formation of γH2AX foci, increased genomic instability and enhanced cellular sensitivity to IR [45].

Several miRNAs were reported to sensitize cells to radiotherapy by inhibiting expression of proteins involved in the HR pathway. A key player in HR, ataxia telangiectasia mutated (ATM), was downregulated by miR-18a in breast cancer, miR-26a in glioma and miR-421 in squamous cell carcinoma (SCC) [19,24,63]. BRCA1, another important protein in HR, was suppressed by miR-182 in breast cancer cells [49]. The HR pathway was also impaired by miR-875 which directly targeted epidermal growth factor receptor (EGFR) and inhibited the EGFR-ZEB1-CHK1 axis. Overexpression of miR-875 enhanced radiosensitivity in prostate cancer cell lines in vitro and in xenograft models through targeting EGFR [70].

Several studies showed a miRNA-mediated regulation of RAD51 expression and the subsequent formation of RAD51 foci in response to IR, an important step in HR. RAD51 was identified as a direct target of miR-34a, miR-107, miR-155 and miR-222 upon IR. Overexpression of miR-34a in lung cancer cells reduced formation of radiation-induced RAD51 foci. This phenotype could be rescued by RAD51 reintroduction. In mouse models administration of MRX34, a liposomal nanoparticle loaded with miR-34a mimics, sensitized lung tumors to radiation by repressing RAD51 [29]. A similar effect was observed for miR-107 and miR-222 mimics in ovarian cancer cells and for overexpression of miR-155 in breast cancer cells [34,48]. Additionally, high miR-155 levels were associated with lower RAD51 expression and better overall survival of patients in a large series of triple-negative breast cancers [48].

### 2.2. lncRNAs

PVT1 was upregulated in patients with nasopharyngeal carcinoma (NPC). PVT1 knockdown enhanced radiosensitivity of NPC cells in vitro and in vivo, which could be attributed to increased apoptosis rate after IR. Decreased phosphorylation of key mediators of DNA damage response, i.e., ATM, p53 and CHK2, was observed in irradiated NPC cells with PVT1 knockdown [131]. This suggests impaired DSB repair upon PVT1 knockdown, however, the level and repair dynamics of IR-induced DSBs was not studied. Wang et al. further showed that PVT1 increased stability of HIF-1α in NPC cells [132]. PVT1 acted as a scaffold for histone acetyltransferase KAT2A, which promoted H3K9 acetylation at the promoter of NF90, a known regulator of HIF-1α expression and stability.

Two lncRNAs, POU6F2-AS2 and DNM3OS, were involved in DSB repair in esophageal squamous cell carcinoma (ESCC). Downregulation of POU6F2-AS2 and DNM3OS promoted radiosensitivity of ESCC cells and impaired DSB repair [111,129]. Analysis of proteins interacting with POU6F2-AS2 revealed among others YBX1, a RNA and DNA binding protein involved in DNA damage response. Ectopic expression of YBX1 partially rescued sensitivity to IR caused by POU6F2-AS2 knockdown, indicating a functional link between POU6F2-AS2 and YBX1 in IR response. Furthermore, it was demonstrated that POU6F2-AS2 is required for YBX1 binding to chromatin, especially to the sites of DNA breaks [129]. DNM3OS knockdown increased the extent of IR-induced DNA damage and impaired DSB repair, as demonstrated by the higher number of γH2AX foci after IR, higher tail moment in comet assay and reduced induction of DNA repair proteins. Interestingly, expression of DNM3OS and radioresistance were promoted by cancer-associated fibroblasts, which are an important component of the tumor environment in ESCC [111].

LINP1 transcripts are localized predominantly in the cytoplasm of Hela S3 cells, but are upregulated and rapidly translocated to the nucleus after IR. Knockdown of LINP1 enhanced radiosensitivity of cervical cancer cells by increasing apoptosis and impairing DSB repair after IR. RNA pulldown revealed association of LINP1 with Ku80 and DNA-PKcs, which suggests that LINP1 is involved in the NHEJ pathway. However, the effect of LINP1 knockdown on Ku80 and DNA-PKcs function was not investigated [122].

Several lncRNAs involved in repair of IR-induced DNA damage interacted with miRNAs. LINC02582 was identified as a direct target of miR-200c in breast cancer, and it promoted radioresistance of breast cancer cells in vitro and in vivo. Upon LINC02582 silencing, the number of irradiation-induced γH2AX foci increased and they persisted longer, which indicated that LINC02582 is involved in DSB repair. Further analysis revealed an interaction of LINC02582 with USP7, a deubiquitinating enzyme stabilizing among others the CHK1 kinase, a crucial player in DNA damage repair. The authors proved that LINC02582 stabilizes CHK1 via USP7 and demonstrated the significance of the miR-200c/LINC02582/USP7/CHK1 axis in radioresistance of breast cancer cells [119].

Other lncRNA–miRNA interactions reported in DNA damage repair include LINC00963 with miR-324-3p and HOTAIR with miR-218 in breast cancer, and MEG3 with miR-182 in thyroid cancer [101,115,118]. miR-218 and miR-182 counteracted the effect of their respective target lncRNAs on DNA damage repair.

## 3. ncRNAs Regulating IR-Induced Apoptosis

**Figure 2:**
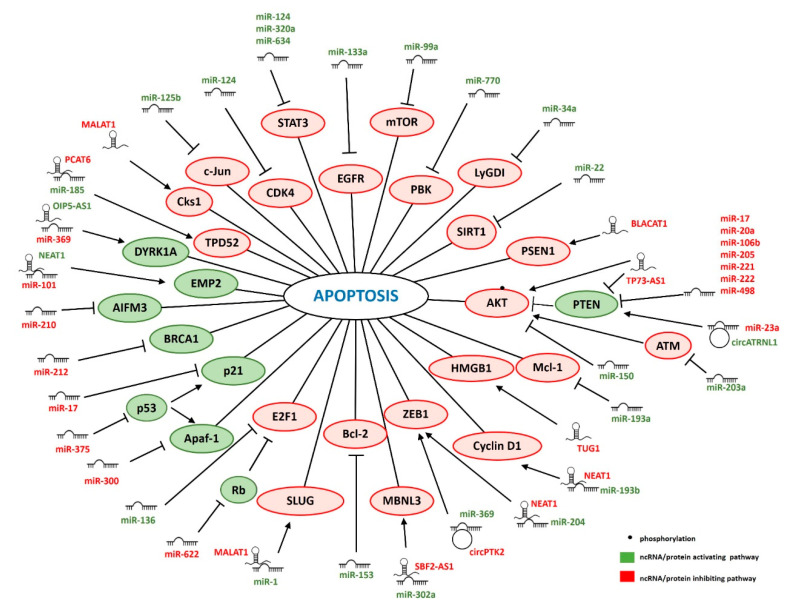
ncRNAs regulating IR-induced apoptosis. ncRNAs promoting (green) or inhibiting apoptosis (red). Pro-apoptotic genes are indicated in green and anti-apoptotic in red.

After exposure to IR, cell death regulatory pathways are activated to eliminate cells with extensive burden of DNA damage [143]. In case repair of IR-induced DNA damage fails, the p53-Induced Death Domain Protein 1 (PIDD) protein is activated in a p53-dependent manner and together with Death Adaptor Molecule RAIDD and caspase-2 this serves as an activation platform for further factors [144,145]. Consequently, cytochrome c is released from damaged mitochondria and activation of caspase-9 triggers a cascade of effector caspases [146]. Several recent studies showed that ncRNAs can modulate programmed cell death after IR [147,148]. Apoptosis is an acclaimed indicator of cellular radiosensitivity and a prognostic factor of radiotherapy treatment outcome [149,150]. Apart from apoptosis, cell death after IR can also occur via necrosis and mitotic catastrophe. However, the evidence for the role of ncRNA in these processes in response to IR in cancer cells is limited. Below we present an overview of the ncRNAs involved in modulation of apoptosis after IR (Figure 2).

### 3.1. miRNAs

Three miRNAs were shown to modulate IR-induced apoptosis by targeting the *TP53* gene or p53 associated proteins. p53 is a crucial tumor suppressor activated in response to IR to induce either cell cycle arrest or apoptosis [151]. miR-375 directly targeted p53. Overexpression of miR-375 reduced p53 expression, enhanced radioresistance, and abrogated p53-mediated apoptosis in IR-treated gastric cancer cells [94]. Apoptotic protease activating factor-1 (Apaf-1), the structural core of the apoptosome, was shown to be directly regulated by miR-300 in lung cancer cells. Ectopic expression of miR-300 caused radioresistance mediated at least in part by a reduction of Apaf-1-induced apoptosis [92]. Wu et al. showed that p21 protein, a target of p53, was downregulated by miR-17 in oral squamous cell carcinoma (OSCC) cells. Suppression of miR-17 in xenograft tumors resulted in increased p21 expression, increased apoptosis rate and enhanced radiosensitivity [72]. miR-210 increased radioresistance of hypoxic hepatoma cells by targeting apoptosis-inducing factor mitochondria-associated 3 (AIFM3) [85]. Downregulation of miR-210 promoted and AIFM3 inhibition attenuated IR-induced apoptosis in hypoxic hepatoma cells. miR-622 was shown to prevent apoptosis by inhibiting the retinoblastoma (Rb) tumor suppressor gene in colorectal cancer cells [96]. The Rb protein can either inhibit or promote apoptosis [152]. The pro-apoptotic role of Rb is associated with Rb-phosphorylation that induces formation of the pRb-E2F1-P/CAF complex. Increased miR-622 levels induced radioresistance, which could be reversed by restoration of Rb. miR-622-mediated Rb inhibition prevented the formation of pRb-E2F1-P/CAF complex [96]. Another miRNA acting on the same pathway, but via targeting E2F1 is miR-136. Similarly to Rb, E2F1 can also induce or inhibit apoptosis depending on the proteins it is associated with [153]. The anti-apoptotic role of E2F1 exerted through the NF-κB signaling pathway was inhibited by miR-136. Overexpression of miR-136 increased radiosensitivity by promoting apoptosis, and inhibition of proliferation in cervical cancer cells [44].

Two miRNAs were shown to affect IR-induced apoptosis by targeting two main anti-apoptotic proteins. Bcl-2 was directly targeted by miR-153 in glioma cells. Levels of miR-153 were lower in radioresistant glioma clinical specimens and glioma cell lines exposed to IR. Overexpression of miR-153 promoted radiosensitivity and apoptosis in glioma cells. This effect was modulated by Bcl-2 inhibition, since restoration of Bcl-2 expression reversed the miR-153-induced phenotype. Additionally, miR-153 enhanced the response to IR in xenograft mice [47]. Another pro-apoptotic miRNA, miR-193a, targeted Mcl-1. Overexpression of miR-193a resulted in increased apoptosis and DNA damage in glioblastoma (GBM) and cervical cancer cells. This effect could be blocked by antioxidant treatment, indicating the crucial role of ROS. Importantly, ectopic expression of Mcl-1 suppressed the pro-apoptotic action of miR-193a, suggesting that Mcl-1 depletion is critical for miR-193a-induced apoptosis [51].

The radiation-inducible miR-770 boosted apoptosis via direct targeting of PDZ-binding kinase (PBK), which sensitized colorectal cancer (CRC) cells to radiation both in vitro and in vivo [69]. Similarly, radiosensitivity and apoptosis were induced by miR-22-mediated inhibition of SIRT1 in breast cancer cells, by miR-34a-mediated inhibition of LyGDI in non-small cell lung cancer (NSCLC) cells and by miR-124-mediated inhibition of CDK4 in ESCC and glioma cells [21,28,35,36]. Moreover, restoration of SIRT and CDK4 reversed the phenotypes [21,35].

Several miRNAs exert their effects on IR response via targeting components of crucial survival pathways, i.e., extracellular signal-regulated kinase (ERK), Janus kinase/signal transducer and activator of transcription (JAK/STAT) and phosphoinositide 3-kinase (PI3K)/AKT. These pathways are initiated in response to IR-dependent activation of EGFR [154]. The ERK pathway was abrogated by the miR-133a-dependent inhibition of EGFR. This led to apoptosis and enhanced radiosensitivity of esophageal cancer cells [42]. Another main player of the ERK pathway, c-Jun is targeted by miR-125b. Forced expression of miR-125b in breast cancer cells resulted in radiosensitivity, enhanced apoptotic activity and senescence upon IR. Restored c-Jun abrogated miR-125b-mediated radiosensitization [41]. STAT3, one of the main players in the JAK/STAT pathway, is a direct target of miR-124, miR-320a and miR-634 [37,60,68]. Overexpression of miR-124 reduced the activity of STAT3 signaling pathway and enhanced apoptosis upon irradiation in NSCLC and in HER2-positive breast cancer [37,38]. In line with these findings, low miR-124 and high STAT3 levels were associated with a poor response to radiotherapy in HER2-positive breast cancer patients [38]. Ectopic expression of miR-320a enhanced IR-induced apoptosis and radiosensitivity of NSCLC cells both in vitro and in vivo [60]. Overexpression of miR-634 promoted apoptosis in irradiated breast cancer cells [68].

High activity of the PI3K/AKT pathway has been associated with resistance to IR-induced apoptosis in many cancer types [155]. AKT was reported to be directly targeted by miR-150 in natural killer (NK)/T-cell lymphoma cells. Overexpression of miR-150 promoted IR-induced apoptosis by suppressing PI3K/AKT signaling and sensitized NK/T-cell lymphoma cells to radiotherapy in a xenograft mouse model [46]. Furthermore, miR-203a-mediated ATM downregulation induced apoptosis and cell cycle arrest in G1 phase in ovarian cancer cells by inhibiting the AKT/GSK3β/Snail signaling pathway [54]. Another miRNA acting through the PI3K/AKT pathway in NSCLC is miR-99a. Ectopic miR-99a expression radiosensitized NSCLC cells in vitro and in vivo by reducing levels of mTOR, one of the downstream targets of AKT kinase [30].

Several miRNAs induce pro-survival signals in response to IR by targeting phosphatase and tensin homolog (PTEN), a primary inhibitor of the PI3K/AKT pathway. The currently known PTEN-targeting miRNAs include miR-17, miR-20a, miR-106b, miR-205, miR-221, miR-222, and miR-498. Overexpression of these miRNAs resulted in activation of the PI3K/AKT pathway, inhibited apoptosis and enhanced radioresistance [73,74,79,83,88,91,95,156]. Another miRNA that attenuated IR-induced apoptosis was miR-212 that directly targeted BRCA1 in glioma cells. Ectopically expressed miR-212 promoted radioresistance which could be phenocopied by BRCA1 knockdown [86].

### 3.2. lncRNAs

Several lncRNAs promoted radioresistance by inhibiting IR-induced apoptosis. Knockdown of lncRNA bladder cancer associated transcript 1 (BLACAT1) accelerated apoptosis of head and neck squamous cell carcinoma cells (HNSCC) by positively regulating expression of presenilin 1 protein (PSEN1). Levels of BLACAT1 and PSEN1 were also positively correlated in HNSCC patients. Overexpression of PSEN1 rescued the enhanced radiosensitivity observed upon BLACAT1 knockdown [105].

Radiation significantly elevated the expression of lncRNA TUG1 in bladder cancer cells. IR-induced apoptosis was noticeably enhanced upon TUG1 silencing and this was due to a downregulation of the anti-apoptotic HMGB1 protein. Restoration of HMGB1 expression reversed the pro-apoptotic effect of TUG1 knockdown [135].

Knockdown of lncRNA TP73-AS1 promoted apoptosis and thereby enhanced radiosensitivity of hepatocellular carcinoma (HCC) cells. This was mediated by upregulated PTEN levels and decreased AKT phosphorylation in HCC cells upon TP73-AS1 silencing. Moreover, knockdown of TP73-AS1 reduced tumor growth in vivo after IR [134].

LncRNA MALAT1 was downregulated after IR in ESCC cells. Overexpression of MALAT1 enhanced the radioresistance of ESCC cells. It was shown that MALAT1 prevented the downregulation of cyclin-dependent kinase subunit (Cks1) after IR, which resulted in a decrease in irradiation-induced apoptosis [123]. MALAT1 and several other lncRNAs also affected IR-induced apoptosis by interacting with miRNAs. In NPC, knockdown of MALAT1 promoted apoptosis and sensitized NPC cells to radiation both in vitro and in vivo. MALAT1 sequestered miR-1 and this resulted in increased levels of the anti-apoptotic SLUG protein [124]. In high risk HPV-positive cervical cancer knockdown of MALAT1 also enhanced IR-induced apoptosis. MALAT1 directly interacted with miR-145 and the combined knockdown of MALAT1 and overexpression of miR-145 had a stronger effect on apoptosis than miR-145 overexpression alone [125]. However, target genes of miR-145 relevant for the effect on apoptosis were not determined.

Contradictory effects of NEAT1 on IR-induced apoptosis were reported [102,126,127]. Wang et al. showed that NEAT1 is downregulated, while Lu et al. observed significantly upregulated levels of NEAT1 in NPC tissues. Wang et al. observed that knockdown of NEAT1 combined with radiation treatment significantly decreased apoptosis in NPC cells. NEAT1 enhanced the expression of the pro-apoptotic epithelial membrane protein 2 (EMP2) by sponging miR-101 [102]. On the contrary, in the study of Lu et al. NEAT1 downregulation reinforced radiosensitivity by enhancing apoptosis of NPC cells. In this case cellular radioresistance was regulated by the NEAT1/miR-204/ZEB1 axis [126]. The opposite effects of NEAT1 on radiosensitivity and IR-induced apoptosis in NPC cells are difficult to reconcile, possibly cell line-specific effects may be involved. In cervical cancer NEAT1 was highly expressed in radioresistant patients. Here, silencing of NEAT1 induced apoptosis in radioresistant cervical cancer cells. NEAT1 reduced miR-193b levels and enhanced expression of Cyclin D1, suggesting that NEAT1 can act as a competing endogenous RNA to sequester miR-193b [127].

Expression of long intergenic non-coding RNA-ROR (lincRNA-ROR) was elevated in CRC cell lines and tissue samples and further induced by IR. Knockdown of lincRNA-ROR enhanced radiosensitivity of CRC cells by increasing apoptosis after IR and it increased expression of p53 and miR-145, and affected the levels of p53/miR-145 targets: p21 and MYC [121].

In contrast to lincRNA-ROR, OIP5-AS1 was downregulated in CRC cell lines. OIP-AS1 dependent downregulation of miR-369 was shown to enhance radiosensitivity and IR-induced apoptosis. Downregulation of miR-369 led to increased levels of DYRK1A [103].

A miRNA-dependent mechanism was also described for lncRNA SBF2-AS1, which affected radiosensitivity of NSCLC through modulating miR-302a and its target MBNL3. Both silencing of SBF2-AS1 and overexpression of miR-302a decreased levels of MBNL3 and promoted apoptosis of NSCLC cells in vitro and in vivo. Furthermore, miR-302a binds to both SBF2-AS1 and MBNL3 indicating that the observed effect of SBF2-AS1 might be caused by sequestering miR-302a. In line with the proposed mechanism, levels of SBF2-AS1 and MBNL3 were elevated, while miR-302a was downregulated in NSCLC tissues from radioresistant patients [133]. However, MBNL3 has not been reported previously to be involved in apoptosis, nor did the current study investigate how MBNL3 affects apoptosis.

PVT1 was another lncRNA promoting radioresistance of NSCLC cells. High levels of PVT1 were negatively correlated with miR-195 expression, and IR further increased PVT1 and reduced miR-195 levels. Direct interaction between PVT1 and miR-195 was demonstrated, and the effect of PVT1 inhibition on radiosensitivity and apoptosis was reversed by inhibition of miR-195 in vitro and in vivo [130]. The targets of miR-195 responsible for the effects on apoptosis remain to be identified.

CCAT1 was upregulated, while miR-148 was downregulated in radioresistant compared to radiosensitive breast cancer tissues. IR further increased CCAT and reduced miR-148 levels. Downregulation of CCAT1 enhanced caspase3 activity and induced apoptosis of breast cancer cells after IR. Direct interaction between miR-148 and CCAT1 was demonstrated, and overexpression of miR-148 copied the effect of CCAT1 knockdown on radiosensitivity and apoptosis of breast cancer cells [106]. However, target genes of miR-148 relevant for apoptosis remain to be determined.

PCAT6 is an oncogenic lncRNA in various cancer types, among others in triple negative breast cancer (TNBC). Knockdown of PCAT6 enhanced radiosensitivity of TNBC cells via induction of apoptosis and G0/G1 cell cycle arrest. Further study revealed that PCAT6 interacted with miR-185 to promote expression of tumor protein D52 (TPD52) [128], an inhibitor of apoptosis overexpressed in several tumors [157].

Two circular RNAs have also been shown to modulate cancer cell radiosensitivity by sequestering miRNAs. circPTK2 was upregulated in gastric cancer tissues. IR further induced expression of circPTK2 in gastric cancer cell lines and its knockdown enhanced radiosensitivity by inducing apoptosis. Moreover, knockdown of circPTK2 reduced tumor growth in vivo. The effect on radiosensitivity and apoptosis was reversed by inhibition of miR-369 which directly interacts with circPTK2. ZEB1 was identified as the relevant target of miR-369, and its ectopic expression restored radioresistance and blocked apoptosis upon miR-369 overexpression [108].

circATRNL1 was downregulated in OSCC tissues. IR further reduced expression of circATRNL1 in OSCC cells, while its ectopic overexpression caused radiosensitization of OSCC cells by inducing apoptosis and promoting cell cycle arrest at G2 phase after IR. This effect was dependent on a direct interaction between circATRNL1 and miR-23a as shown by phenotype rescue experiments using miR-23a mimics. This was mediated by the miR-23a target gene PTEN which inactivates the AKT signaling pathway [98].

## 4. ncRNAs Involved in IR-Related Autophagy

**Figure 3:**
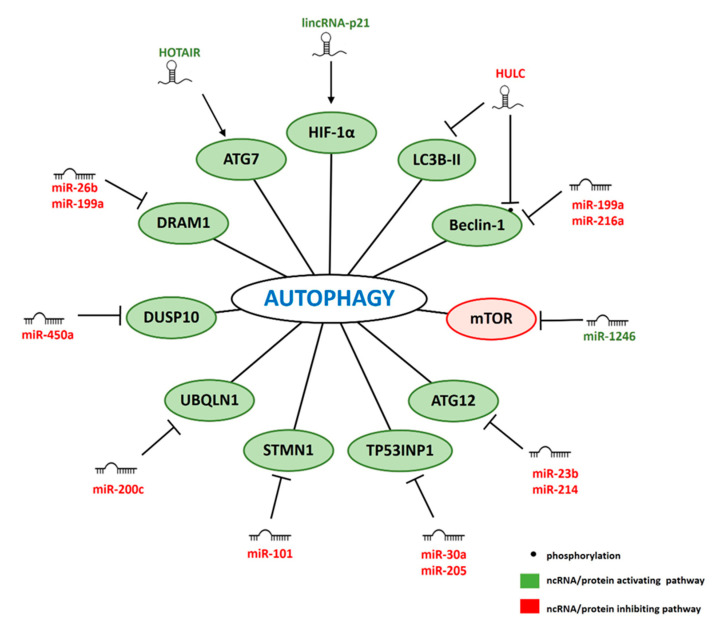
ncRNAs regulating IR-induced autophagy. ncRNAs and proteins promoting (green) or inhibiting (red) autophagy.

In normal conditions autophagy is a well-controlled survival mechanism helping to recycle damaged proteins and cellular organelles. Induction of autophagy leads to formation of double-membrane vesicles called autophagosomes. This multistep process is controlled by a number of proteins, i.a. Beclin-1, LC3B-II, mTOR, and autophagy-related proteins (ATG). After fusion of the autophagosome with endosome and lysosome, the autolysosome is formed [158]. Then the autolysosome containing cytoplasm-derived contents is degraded together with its inner membrane by lysosomal hydrolases [158,159]. Autophagy induced by IR plays a crucial role in determining cell fate: whether cells survive or die and it also affects radiosensitivity [160]. On one hand, autophagy induced by radiation has a cytoprotective function allowing the cell to eliminate toxic species [161,162]. On the other hand, autophagy can enhance the anticancer effects of some drugs and serve as additional cell death pathway [162,163,164]. Below we present an overview of the ncRNAs currently known to be involved in regulation of IR-induced autophagy (Figure 3).

### 4.1. miRNAs

Two miRNAs directly regulate Beclin-1, a central regulator of autophagy that controls autophagosome nucleation and maturation [165]. Forced expression of miR-216a in radioresistant pancreatic cancer cells enhanced radiosensitivity by inhibiting autophagy in response to irradiation. These effects could be abrogated by overexpression of Beclin-1. Furthermore, miR-216a overexpression sensitized xenograft tumor to irradiation treatment by inhibiting IR-induced autophagy via targeting Beclin-1 [56]. Yi et al. showed that miR-199a mimic suppressed IR-induced autophagy in MCF7 breast cancer cells by targeting Beclin-1 and DNA damage-regulated autophagy modulator protein 1 (DRAM1) [52]. DRAM1 is also targeted by miR-26b in breast cancer cells and both overexpression of miR-26b and inhibition of DRAM1 reduced IR-induced autophagy [25].

miR-23b inhibited IR-induced autophagy by targeting ATG12, an ubiquitin-like protein involved in autophagy vesicles formation. Overexpression of miR-23b inhibited autophagy and sensitized pancreatic cancer cells to radiation. This effect was also confirmed in a xenograft model upon IR. In line with this, reduced levels of miR-23b resulted in increased levels of ATG12 and increased autophagy were observed in radioresistant compared to radiosensitive pancreatic cancer cells. Additionally, miR-23b expression inversely correlated with autophagy activity in human pancreatic cancer tissues [22]. Similarly, Hu et al. showed that miR-214 promoted radiosensitivity by targeting ATG12 and inhibiting IR-induced autophagy in CRC both in vitro and in vivo [55].

Several other miRNAs have been shown to inhibit IR-induced autophagy by targeting autophagy activators. MiR-200c targeted Ubiquilin-1 (UBQLN1), a promoter of autophagosome formation [166]. Ectopic expression of miR-200c sensitized breast cancer cells to irradiation via inhibition of autophagy. Inverse correlation between the levels of miR-200c, UBQLN1 and autophagy activity was observed in human breast cancer tissues [53]. Another autophagy activator, Stathmin 1 (STMN1) was identified as miR-101 target in NPC cells. Ectopic expression of miR-101 suppressed radiation-induced autophagy of NPC cells. This effect was reversed by STMN1 restoration [33]. A similar effect was shown for inhibition of Tumor protein 53-induced nuclear protein 1 (TP53INP1) by miR-30a and miR-205 in prostate cancer cells [26]. MiR-450a targeted DUSP10 and this impaired IR-induced autophagy by affecting the ERK pathway and regulating ROS elimination. Additionally, tumor xenograft experiments verified that miR-450a overexpression could increase sensitivity to radiotherapy in radioresistant ESCC cells in vivo [67].

One miRNA that promoted autophagy in NSCLC cells was miR-1246. Ectopic expression of miR-1246 downregulated mTOR and decreased radiosensitivity of lung cancer cells both in vitro and in vivo. Overexpression of mTOR reversed the effect [97].

### 4.2. lncRNAs

lincRNA-p21 was upregulated after IR and hypoxia treatment in hepatoma and glioma cells lines. The number of autophagic vesicles upon lincRNA-p21 knockdown was significantly decreased in hypoxic tumor cells. This effect was mediated by downregulation of HIF-1α and activation of the AKT/mTOR/p70S6K pathway. Moreover, overexpression of HIF-1α abolished the radiosensitizing effect of lincRNA-p21 knockdown [120].

HULC regulates sensitivity of prostate cancer cells to IR by modulating microtubule-associated protein 1 light chain 3B II (LC3B-II), which plays an essential role in the formation of autophagosome. Overexpression of HULC decreased, and its knockdown increased, LC3B-II expression after IR. Moreover, HULC can bind to Beclin-1 and increase its phosphorylation at threonine 119 [117]. This leads to dissociation of Beclin-1 from the complex with Bcl-2 protein and in turn results in activation of autophagy [165], explaining the mode of action of HULC in promoting autophagy [117].

Another lncRNA linked to autophagy is HOTAIR. HOTAIR promoted radiosensitivity of pancreatic cancer cells by increasing the levels of LC3-II. This indicated that HOTAIR can promote autophagosome formation. Moreover, HOTAIR enhanced expression of ATG7, a key protein involved in autophagosome formation that is responsible for vesicle progression [116]. In line with this, Guo et al. demonstrated that HOTAIR knockdown inhibited autophagy through suppression of the Wnt signaling pathway in radioresistant human cervical cancer HeLa cells [167].

## 5. ncRNAs Regulating Cell Cycle in Response to IR

**Figure 4:**
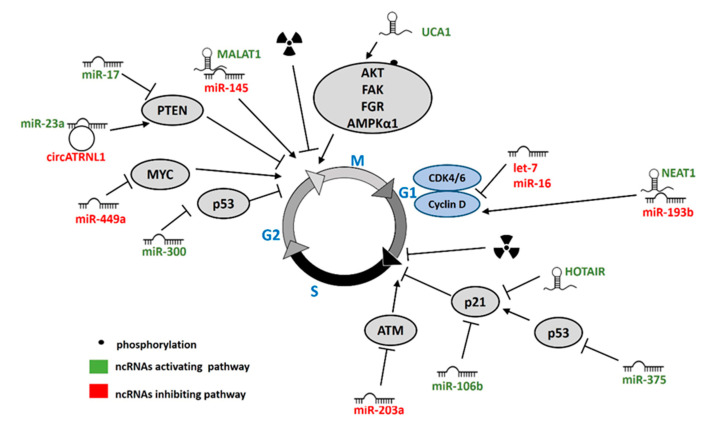
ncRNAs regulating IR-induced cell cycle. ncRNAs promoting (green) or inhibiting (red) cell cycle progression.

In normal cells IR delays progression through the G1, S, and G2 phases of the cell cycle to allow for DNA repair and to prevent accumulation of harmful genomic lesions. The central regulator of cell cycle progression is p53, whose phosphorylation by ATM induces expression and phosphorylation of the cyclin-dependent kinase inhibitor p21. This leads to inhibition of CDK4/6-cyclin D and CDK1-cyclin B, causing cell cycle arrest in G1 and G2, respectively [168]. In addition, activation of signal transducers CHK1 and CHK2 by ATM and ATR promotes degradation of CDC25, leading to inhibition of CDK2-cyclin E and CDK1-cyclin B, which promotes cell cycle arrest in G1 and G2, respectively [169]. Effective induction of cell cycle arrest promotes radiosensitivity which means that the ability to progress through cell cycle after IR contributes to tumor radioresistance [170]. Several ncRNAs have been shown to play a role in cell cycle regulation after IR (Figure 4).

### 5.1. miRNAs

Several miRNAs affect cell cycle arrest and as such play a role in radiosensitivity. Cyclin D1 was identified as a target gene of let-7 and miR-16. Overexpression of let-7b resulted in cyclin D1 downregulation and sensitization of uveal melanoma cells to irradiation by increasing G1 arrest in vitro and in vivo [16]. Overexpression of miR-16 induced G0/G1 phase arrest and suppressed cell proliferation after IR resulting in enhanced radiosensitivity of prostate cancer cells [18]. MiR-375 abrogates the cell cycle G1 arrest through inactivation of the p53 pathway in gastric cancer cells [94]. Additionally, miR-300 desensitized lung cancer cells to IR by suppressing p53-dependent G2 cell cycle arrest [92]. Overexpression of miR-145 increased radiosensitivity of cervical cancer cells and induced G2/M phase block [125]. CDK inhibitor p21 and anti-proliferation protein PTEN were both directly targeted by miR-106b in colorectal cancer cells. Overexpression of miR-106b induced radioresistance in CRC cells by promoting G1 to S transition, which was abrogated by overexpression of either PTEN or p21 [79]. Similar to miR-106b, miR-17-mediated PTEN inhibition promoted G2 to M progression and enhanced proliferation of NPC cells. The effect of miR-17 overexpression was mediated by AKT signaling, as shown by reversion upon treatment with an AKT inhibitor [79]. Inhibition of c-MYC by miR-449a resulted in IR-induced G2/M arrest in prostate cancer cells. This effect was mediated by abrogated control of Cdc2/Cyclin B1 signaling. Additionally, both overexpression of miR-449a and knockdown of c-MYC sensitized prostate cancer cells to IR and miR-449a also enhanced radiosensitivity in xenograft models [66].

### 5.2. lncRNAs

HOTAIR was shown to regulate several aspects of cellular response to IR, amongst them cell cycle. In cervical cancer knockdown of HOTAIR induced G1 phase arrest and thereby increased radiosensitivity in vitro and in vivo. G1 arrest upon HOTAIR knockdown was reversed by p21 inhibition. These findings demonstrate that HOTAIR regulates cell cycle progression in response to IR by inhibiting p21. In line with these results, HOTAIR was negatively correlated with p21 levels in cancer tissues [113].

Two other lncRNAs were involved in cell cycle regulation in cervical cancer: MALAT1 and NEAT1. High levels of MALAT1 characterized radioresistant cases of cervical cancer, and IR induced MALAT1 expression in cervical cancer cell lines. Knockdown of MALAT1 enhanced radiosensitivity by inducing apoptosis and G2/M arrest [126]. NEAT1 was also more abundant in radioresistant cases of cervical cancer. Similarly to MALAT1, knockdown of NEAT1 enhanced radiosensitivity by inducing apoptosis and cell cycle arrest in G1 phase. Silencing of Cyclin D1 copied the effect of NEAT1 inhibition on radioresistance, cell cycle and apoptosis. On the other hand, Cyclin D1 overexpression or inhibition of miR-193b, which targets Cyclin D1, partially rescued the phenotype of NEAT1 inhibition. This showed that the effect of NEAT1 in response to IR is at least in part dependent on miR-193b and Cyclin D1. However, in vivo experiments demonstrated that although knockdown of NEAT1 reduced tumor volume and weight, combination with IR did not result in further reduction, and the observed effect was even worse than radiotherapy alone. This discrepancy between in vitro and in vivo models requires further clarification [127].

lncRNA UCA1 promoted radioresistance in colorectal and prostate cancer. UCA1 inhibition rendered prostate cancer cells more sensitive to IR and resulted in accumulation of cells in the G2/M phase. Mechanistically, UCA1 promoted phosphorylation of several kinases implicated in tumor radioresistance and cell cycle regulation: AKT, FAK, FGR, and AMPKα1 [136]. Unlike in prostate cancer, UCA1 inhibition in CRC cells attenuated the G2/M arrest induced by IR, while it promoted apoptosis and inhibited migration and EMT of CRC cells [171]. Thus, the mechanism underlying UCA1 effect on radioresistance involves cell cycle regulation in prostate cancer, but apparently not in CRC.

## 6. ncRNAs Modulating Glycolysis in IR-Response

**Figure 5:**
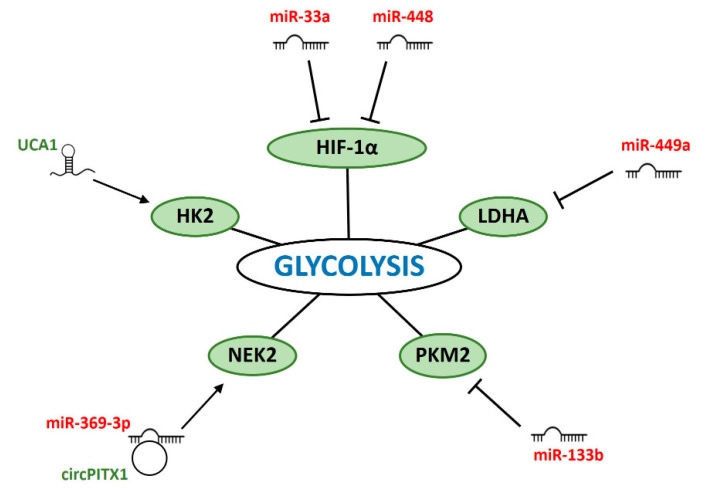
ncRNAs regulating IR-induced glycolysis. ncRNAs promoting (green) or inhibiting (red) glycolysis.

Cancer cells exhibit increased energy demand to sustain their rapid proliferation and growth. The energy mostly derives from glycolysis, a process in which glucose is converted to pyruvate, providing energy and intermediates for other metabolic pathways. This preference of cancer cells for anaerobic glycolysis over oxidative phosphorylation is recognized as the Warburg effect [172]. Several glycolytic enzymes, e.g., HK2, LDHA, GLUT1, and PKM2 are upregulated in cancer cells [173]. Reprogramming of glucose metabolism in cancer cells is driven by HIF-1α and c-Myc [174]. Glycolysis has a major contribution to radioresistance of cancer cells, and targeting glycolytic enzymes has been shown to improve radiotherapy efficacy [175]. A number of ncRNAs are involved in determining radiosensitivity of cancer cells by regulating glycolysis (Figure 5).

### 6.1. miRNAs

Two miRNAs, miR-33a and miR-448, directly targeted HIF-1α to inhibit glycolysis and promote radiosensitivity of cancer cells. Overexpression of miR-33a enhanced radiosensitivity of melanoma cells in vitro and in vivo by inhibiting enzymes crucial for glycolysis: HIF-1α, HK1, HK2, and LDHA. Overexpression of HIF-1α, a previously identified miR-33a target [176], rescued the miR-33a-mediated inhibition of glycolysis [27]. A similar mechanism was reported for miR-448 in glioma cells. miR-448 directly targeted HIF-1α, which led to inhibition of proteins involved in glycolysis: HK1, HK2, LDHA [64].

miR-133b sensitized NSCLC cells to IR and negatively regulated glycolysis. This effect was mediated by targeting PKM2, an enzyme converting P-enolpyruvate to pyruvate. Overexpression of PKM2 rescued the effect of miR-133b on radiosensitivity and glycolysis [43]. Another miRNA that inhibited glycolysis, miR-449a, was shown to target LDHA, an enzyme which catalyzes the last step in glycolysis, conversion of pyruvate to lactate. miR-449a mimics promoted radiosensitivity of lung cancer cells, resulting in enhanced apoptosis and DNA damage, and reduced glycolysis rate. Silencing of LDHA copied the effect of miR-449 overexpression on cellular response to IR [65].

### 6.2. lncRNAs

In addition, a lncRNA and a circRNA have been implicated in regulating glycolysis in response to IR. Knockdown of lncRNA UCA1 enhanced radiosensitivity and reduced glycolysis rates in cervical cancer cell lines by decreasing levels of HK2, a key enzyme involved in the first step of glycolysis. Treatment with inhibitor of glycolysis reversed the positive effect of UCA1 overexpression on HK2 levels and radioresistance [137]. Silencing of circPITX1 promoted radiosensitivity of glioma cells in vitro and in vivo by inhibiting glycolysis. circPITX1 acted as a sponge for miR-329-3p to protect NEK2 from miRNA-mediated inhibition. The relevance of the circPITX1/miR-329-3p/NEK2 axis in radioresistance and glycolysis was confirmed by rescue experiments and inhibition of glycolysis [107]. Although not directly involved in glycolysis, NEK2 was previously shown to regulate splicing of pyruvate kinase M2 (PKM2), a key player in this pathway [177].

## 7. ncRNAs Affecting the Wnt/β-Catenin Pathway in Response to IR

**Figure 6:**
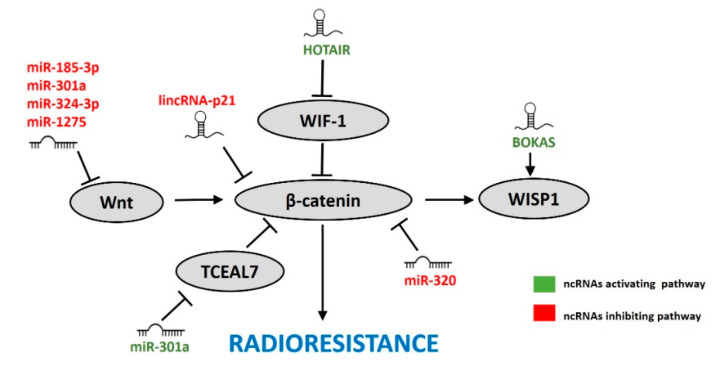
ncRNAs involved in the IR-induced Wnt/β-catenin pathway. ncRNAs promoting (green) or inhibiting (red) the Wnt/β-catenin pathway-induced radioresistance.

The Wnt/β-catenin signaling is an example of an oncogenic pro-survival pathway hyperactivated in many types of cancer [178,179] and promotes reprogramming of cancer cell metabolism and cancer immunity [180]. Recently, the Wnt/β-catenin pathway has also been implicated in stimulating radioresistance of cancer cells by affecting cell cycle, apoptosis, DNA repair, and cell proliferation [181]. Several miRNAs and lncRNAs have also been implicated in the Wnt/β-catenin pathway (Figure 6).

### 7.1. miRNAs

miR-320 directly targeted β-catenin in cervical cancer cells. Both overexpression of miR-320 and knockdown of β-catenin promoted radiosensitivity of cervical cancer cells. In line with this, miRNA-320 levels were decreased and β-catenin levels increased in radioresistant cervical cancer cells compared to more sensitive parental cells [61].

Contradictory effects of miR-301a on Wnt/β-catenin signaling pathway and radiosensitivity were demonstrated. miR-301a was shown to directly target Wnt that activates and TCEAL7 that suppress β-catenin signaling [58,93]. Upregulation of miR-301a directly inhibited Wnt and indirectly β-catenin and cyclin D1 in ESCC cells and this led to increased radiosensitivity, reduced proliferation and migration of ESCC cells [58]. In contrast, miR-301a-mediated TCEAL7 inhibition increased transcriptional activity of β-catenin. Moreover, inhibition of miR-301a and overexpression of TCEAL7 reversed the radioresistant miR-301a phenotype in GMB cells in vitro and in vivo [93]. These opposite effects could be attributed to a cell-line specific miR-301a function.

miR-185-3p sensitized NPC cells to IR by targeting Wnt2B protein and an inverse relationship between expression levels of miR-185-3p and Wnt2B was confirmed in NPC cells and tissues [50]. Wnt2B is also targeted by miR-324-3p, whose overexpression increased radiosensitivity and impaired Wnt2B signaling after IR. Furthermore, miR-324-3p levels decreased and Wnt2B levels increased in NPC tissues after radiotherapy [62]. In addition, miR-1275 directly targeted Wnt1 in ESCC cells. Overexpression of miR-1275 enhanced radiosensitivity via inhibition of the epithelial to mesenchymal transition (EMT). Moreover, miR-1275 reduced tumor growth and promoted radiosensitivity in vivo [71].

### 7.2. lncRNAs

A handful of lncRNAs have been linked to IR response by regulating the Wnt/β-catenin pathway. lincRNA-p21 was downregulated in gastric cancer and CRC cells and tissues [99,100]. Ectopic overexpression of lincRNA-p21 enhanced radiosensitivity and inhibited the Wnt/β-catenin pathway in both tumor types. Levels of β-catenin and its target genes, c-MYC and Cyclin D1, were also downregulated upon lincRNA-p21 overexpression, as well as upon IR in CRC [100]. Inhibition of the β-catenin pathway abolished the effects of lincRNA-p21 overexpression on cell proliferation and radiosensitivity in gastric cancer cells [99]. This supports the role of lincRNA-p21 in promoting radiosensitivity by inhibiting the Wnt/β-catenin pathway.

In pancreatic ductal adenocarcinoma (PDAC) cells HOTAIR was induced by IR, while its knockdown enhanced radiosensitivity. Upon HOTAIR knockdown expression of Wnt inhibitory factor 1 (WIF-1) was increased and in line with that, levels of HOTAIR and WIF-1 were negatively correlated in clinical samples. Furthermore, overexpression of WIF-1 enhanced radiosensitivity of PDAC cells, copying the effect of HOTAIR knockdown [114]. Although the activity of Wnt/β-catenin pathway was not assessed in these experiments, the authors suggest that HOTAIR may be involved in IR response in PDAC cells by promoting this pathway.

WISP1 is a downstream target of Wnt/β-catenin pathway. It was upregulated in ESCC tissues and promoted radioresistance of ESCC cells in vitro and in vivo. Knockdown of lncRNA BOKAS, an IR-induced lncRNA, reduced expression of WISP1 upon IR [104]. However, it was not demonstrated whether BOKAS affects WISP1 via the Wnt/β-catenin pathway or if some other mechanism is involved.

## 8. Conclusions

Non-coding RNAs have emerged as key regulators in various processes in health and disease. In cancer, ncRNAs have been shown to contribute to carcinogenesis, differentiate between cancer subtypes, predict prognosis, and influence treatment outcome. Radiation is a treatment of choice for many cancer types. However, while some tumors are sensitive to radiotherapy, others demonstrate radioresistance. Therefore, mechanisms underlying tumor radioresistance and potential options of combination therapy to improve the outcome are being investigated. Here, we have summarized the current knowledge on the role of ncRNAs in determining the radiosensitivity of various cancers. From this review, it is evident that miRNAs and lncRNAs are involved in many aspects of cellular response to radiation. While some promote tumor radiosensitivity, others render tumors more radioresistant. Expression levels of several of these ncRNAs are also affected by IR. One of the key players in IR response, p53, regulates the expression of several miRNAs (e.g., miR-34 [182]) and lncRNAs (e.g., Damage Induced Noncoding RNA (DINO) [183]), and can itself be regulated by ncRNAs [182,184]. Many ncRNAs have been associated with response to radiotherapy and can be used to predict efficacy of radiation treatment. Functional studies revealed different underlying mechanisms, involving crucial aspects of cellular response to IR, such as DNA damage repair, cell cycle control, apoptosis etc. Moreover, in vitro and in vivo studies demonstrated that modulation of the levels of ncRNAs can significantly enhance radiosensitivity of tumor cells. This indicates that ncRNAs may be used as targets to improve outcome of radiotherapy. The functional relevance was shown for multiple ncRNAs, indicating a great potential in combination radiotherapy. In recent years, several preclinical and clinical trials targeting ncRNAs, especially miRNAs, have been initiated. This further demonstrates the therapeutic potential of targeting RNA. With the development of advanced delivery strategies for RNA-based therapeutics, new combined radio-RNA therapies can emerge. Next to this, more studies are needed to identify ncRNAs serving as biomarkers of response to radiation in different cancer types. Further functional studies to determine underlying mechanisms of ncRNAs promoting radiosensitivity or radioresistance, as well as confirmation using in vivo models are also warranted.

## Figures and Tables

**Table 1:** microRNAs (miRNAs) promoting radiosensitivity or radioresistance in cancer cells.

miRNA	Target	Pathway	Cancer Type	Reference
**miRNA Promoting Radiosensitivity**				
let-7	Cyclin D1	Cell cycle	Uveal melanoma *, breast cancer	[16,17]
miR-16	Cyclin D1	Cell cycle	Prostate cancer	[18]
miR-18a	ATM	DSB repair	Breast cancer, lung cancer *	[19,20]
	HIF-1α	-	Lung cancer *	[20]
miR-22	SIRT1	Apoptosis	Breast cancer	[21]
miR-23b	ATG12	Autophagy	Pancreatic cancer *	[22]
miR-24	H2AX	DSB repair	Leukemia	[23]
miR-26a	ATM	DSB repair	Glioma	[24]
miR-26b	DRAM1	Autophagy	Breast cancer	[25]
miR-30a	TP53INP1	Autophagy	Prostate cancer	[26]
miR-33a	HIF-1α	Glycolysis	Melanoma *	[27]
miR-34a	LyGDI	Apoptosis	NSCLC	[28]
	RAD51	DSB repair	Lung cancer *	[29]
miR-99a	mTOR	Apoptosis	NSCLC *	[30]
miR-100	ATM	-	Glioma	[31]
miR-101	DNA-PKcs	-	Glioma *	[32]
	ATM	-	Glioma *	[32]
	STMN1	Autophagy	NPC	[33]
miR-107	RAD51	DSB repair	Ovarian cancer	[34]
miR-124	CDK4	Apoptosis	Glioma, ESCC	[35,36]
	STAT3	Apoptosis	NSCLC *, breast cancer	[37,38]
	PIM 1	-	Prostate cancer	[39]
miR-125a	p21	-	Cervical cancer	[40]
miR-125b	c-Jun	Apoptosis	Breast cancer	[41]
miR-133a	EGFR	Apoptosis	ESCC	[42]
miR-133b	PKM2	Glycolysis	NSCLC	[43]
miR-136	E2F1	Apoptosis	Cervical cancer	[44]
miR-138	H2AX	DSB repair	Osteosarcoma	[45]
miR-144	PIM 1	-	Prostate cancer	[39]
miR-150	AKT	Apoptosis	NK/T cell lymphoma *	[46]
miR-153	Bcl-2	Apoptosis	Glioma *	[47]
miR-155	RAD51	DSB repair	Triple negative breast cancer *	[48]
miR-182	BRCA1	DSB repair	Breast cancer	[49]
miR-185	Wnt2B	Wnt/β-catenin	NPC	[50]
miR-193a	Mcl-1	Apoptosis	GBM, cervical cancer	[51]
miR-199a	Beclin-1, DRAM1	Autophagy	Breast cancer	[52]
miR-200c	UBQLN1	Autophagy	Breast cancer	[53]
miR-203a	ATM	Apoptosis, cell cycle, migration	Ovarian cancer	[54]
miR-205	TP53INP1	Autophagy	Prostate cancer	[26]
miR-214	ATG12	Autophagy	CRC *	[55]
miR-216a	Beclin-1	Autophagy	Pancreatic cancer *	[56]
miR-222	RAD51	DSB repair	Ovarian cancer	[34]
miR-223	ATM	-	Glioma *	[57]
miR-301a	Wnt	Wnt/β-catenin, migration	ESCC	[58]
miR-302a	AKT, RAD52	-	Breast cancer *	[59]
miR-320a	STAT3	Apoptosis, metastasis	NSCLC *	[60]
	β-catenin	Wnt/β-catenin	Cervical cancer	[61]
miR-324-3p	Wnt2B	Wnt/ β-catenin	NPC	[62]
miR-421	ATM	DSB repair	SCC	[63]
miR-448	HIF-1α	Glycolysis	Glioma	[64]
miR-449a	LDHA	Glycolysis, apoptosis	Lung cancer	[65]
miR-449a	c-MYC	Cell cycle	Prostate cancer *	[66]
miR-450a	DUSP10	Autophagy	ESCC *	[67]
miR-634	STAT3	Apoptosis	Breast cancer	[68]
miR-770	PBK	Apoptosis	CRC *	[69]
miR-875	EGFR	DSB repair	Prostate cancer *	[70]
miR-1275	Wnt1	Wnt/β-catenin, EMT,	ESCC *	[71]
**miRNA Promoting Radioresistance**				
miR-17	P21	Apoptosis	OSCC *	[72]
	PTEN	Apoptosis, cell cycle	NPC	[73]
miR-20a	PTEN	Apoptosis	HCC	[74]
miR-21	PTEN	Migration	Leukemia, NSCLC	[75,76]
miR-29a	PTEN	-	CRC	[77]
miR-96	PTEN	-	HNSCC	[78]
miR-106b	PTEN, p21	Apoptosis, cell cycle	CRC	[79]
miR-135b	GSK3β	-	GBM	[80]
miR-150	GSK3β	-	NPC	[81]
miR-155	UBQLN1	-	NPC	[82]
miR-205	PTEN	Apoptosis	NPC	[83]
miR-208a	p21		Lung cancer	[84]
miR-210	AIFM3	Apoptosis	Hepatoma	[85]
miR-212	BRCA1	Apoptosis	Glioma	[86]
miR-214	PTEN	-	Ovarian cancer *	[87]
miR-221/miR-222	PTEN	Apoptosis, migration	CRC, gastric cancer, GBM	[88,89,90,91]
miR-300	p53, Apaf-1	Apoptosis, cell cycle, senescence	Lung cancer	[92]
miR-301a	TCEAL7	Wnt/β-catenin, apoptosis	GBM *	[93]
miR-375	p53	Apoptosis, cell cycle	Gastric cancer	[94]
miR-498	PTEN	Apoptosis, migration	Prostate cancer	[95]
miR-622	Rb	Apoptosis	CRC	[96]
miR-1246	mTOR	Autophagy	NSCLC	[97]

* Effect on radiosensitivity/radioresistance observed in vivo; HNSCC—head and neck squamous cell carcinoma; HCC—hepatocellular carcinoma; NPC—nasopharyngeal carcinoma; CRC—colorectal cancer; NSCLC—non-small cell lung cancer; OSCC—oral squamous cell carcinoma; ESCC—esophageal squamous cell carcinoma; GBM—glioblastoma; NK/T-cell lymphoma—natural killer/T-cell lymphoma; DSB—DNA double strand break.

**Table 2:** Long non-coding RNAs (lncRNAs) promoting radiosensitivity or radioresistance in cancer cells.

lncRNA	Target	Pathway	Cancer Type	Reference
**lncRNA Promoting Radiosensitivity**				
circATRNL1	miR-23a/PTEN	Apoptosis, cell cycle	OSCC	[98]
lincRNA-p21	β-catenin	Wnt/β-catenin	Gastric cancer	[99]
	-	Wnt/β-catenin	CRC	[100]
MEG3	miR-182	DSB repair	Thyroid cancer	[101]
NEAT1	miR-101/EMP2	Apoptosis, EMT	NPC *	[102]
OIP5-AS1	miR-369/DYRK1A	Apoptosis	CRC	[103]
**lncRNA Promoting Radioresistance**				
BOKAS	WISP1	Wnt/β-catenin	ESCC	[104]
BLACAT1	PSEN1	Apoptosis, cell cycle DSB repair	HNSCC	[105]
CCAT1	miR-148	Apoptosis	Breast cancer	[106]
circPITX1	miR-329-3p/NEK2	Glycolysis	Glioma *	[107]
circPTK2	miR-369/ZEB1	Apoptosis	Gastric cancer *	[108]
CRNDE	-	Apoptosis	Lung adenocarcinoma	[109]
CYTOR	-	Apoptosis	NSCLC	[110]
DNM3OS	-	DSB repair	ESCC *	[111]
FAM83H-AS1	HuR	DSB repair	Ovarian cancer	[112]
HOTAIR	p21	Apoptosis, cell cycle	Cervical cancer*	[113]
	WIF-1	Wnt/β-catenin	Pancreatic cancer	[114]
	miR-218	Apoptosis, cell cycle, DSB repair	Breast cancer	[115]
	LC3-II, ATG7	Autophagy, apoptosis	Pancreatic cancer	[116]
HULC	LC3B-II, Beclin-1	Apoptosis, autophagy	Prostate cancer	[117]
LINC00963	miR-324-3p/ACK1	DSB repair	Breast cancer	[118]
LINC02582	miR-200c/USP7/CHK1	DSB repair	Breast cancer *	[119]
lincRNA-p21	HIF-1α	Autophagy	Hepatoma, Glioma	[120]
lincRNA-ROR	P53, miR-145	Apoptosis	CRC *	[121]
LINP1	Ku80, DNA-PKcs	Apoptosis, DSB repair	Cervical cancer	[122]
MALAT1	Cks1	Apoptosis	ESCC *	[123]
	miR-1/SLUG	Apoptosis	NPC *	[124]
	miR-145	Apoptosis, cell cycle	Cervical cancer	[125]
NEAT1	miR-204/ZEB1	Apoptosis	NPC	[126]
	miR-193b/Cyclin D1	Apoptosis, cell cycle	Cervical cancer *	[127]
PCAT6	miR-185/TPD52	Apoptosis, cell cycle	Breast cancer	[128]
POU6F2-AS2	YBX1	DSB repair	ESCC	[129]
PVT1	miR-195	Apoptosis, DSB repair	NSCLC *	[130]
	ATM, p53, CHK2	Apoptosis, DSB repair	NPC *	[131]
	HIF-1α	DSB repair	NPC	[132]
SBF2-AS1	miR-302a/MBNL3	Apoptosis	NSCLC *	[133]
TP73-AS1	PTEN, AKT	Apoptosis	HCC *	[134]
TUG1	HMGB1	Apoptosis	Bladder cancer *	[135]
UCA1	AKT, FAK, FGR, AMPKα1	Cell cycle	Gastric cancer	[136]
UCA1	HK2	Glycolysis	Cervical cancer	[137]

* Effect on radiosensitivity/radioresistance observed in vivo; HNSCC—head and neck squamous cell carcinoma; HCC—hepatocellular carcinoma; NPC—nasopharyngeal carcinoma; CRC—colorectal cancer; NSCLC—non-small cell lung cancer; OSCC—oral squamous cell carcinoma; ESCC—esophageal squamous cell carcinoma; lincRNA—long intergenic noncoding RNA.
